# The future of healthcare has arrived: who dares take up the challenge?

**DOI:** 10.1007/s12471-018-1204-3

**Published:** 2018-12-10

**Authors:** O. Gerrits

**Affiliations:** Health Procurement, Zilveren Kruis, Leusden, The Netherlands

**Keywords:** e-Health, Telemonitoring, Health insurer, Zilveren Kruis, Heart failure, Quality

## Abstract

According to the Euro Health Consumer Index, the Dutch healthcare system is the champion of Europe. Yet we are living for longer, prosperity is increasing and the population is growing. If we do not continue to adjust our healthcare system to these trends, medical expenses in the Netherlands will double to € 174 billion (in 2040). We are also facing job market difficulties in healthcare. We risk a shortfall of 125 thousand employees in 2022. It is therefore time to restructure healthcare. Not tomorrow but today. Healthcare will increasingly be organised around the day-to-day lives of patients—at home or work, with offline and online healthcare seamlessly matching up to each other. The shortage of personnel also demands a different attitude to healthcare provision. As a health insurer we can help to improve healthcare, for instance by giving healthcare providers the financial capacity to organise their care differently. Or by conducting independent research so that conclusions can be drawn on the legal criterion of ‘the state of science and practice’. This is how it was possible, in conjunction with the Dutch Cardiology Centres, the Netherlands Society of Cardiology and FocusCura, to include the ‘Hartwacht’ telemonitoring service in health insurance policies.

According to the Euro Health Consumer Index, the Dutch healthcare system is the champion of Europe. Scores for aspects such as accessibility, the outcome of care and patient rights clearly demonstrate this. Yet we are living for longer, prosperity is increasing and the population is growing. If we do not continue to adjust our healthcare system to these trends, medical expenses in the Netherlands will double to € 174 billion in 2040. We are also facing growing job market difficulties in healthcare. If we fail to act, we risk a shortfall of 100 to 125 thousand employees in 2022. These forecasts are very clear too. It is therefore time to restructure healthcare—not tomorrow but today.

## New way of thinking, use of new resources

Restructuring healthcare requires a new way of thinking and the use of new resources. Healthcare will increasingly be organised around the day-to-day lives of patients—at home or work, with offline and online healthcare seamlessly matching up to each other. e‑Health will play an ever more prominent role in this transition. The shortage of personnel also demands a different attitude to healthcare provision. The emphasis will increasingly be on encouraging self-reliance rather than taking over. The ability of patients to take control and the power of digital resources will be used to the full here.

## We are heading in the right direction, all we need now is to get up to speed

The shift of healthcare to the home has been ongoing for a while now. Although our life expectancy is rising ever higher, the number of hospital admissions has stayed the same over the past 35 years [1]. This is a step-by-step shift and one that requires adjustments to healthcare processes and how parties work together in order to develop healthcare services close to people’s homes and reduce them in hospitals. It also demands investment in digital parameters and upscaling of innovations in order to bring down expenses. Yet the current rate of change is too slow to effect the cost restructuring that will enable us to keep the increase in medical expenses manageable. From health insurance companies to specialists, right up to supervisory authorities, all the parties involved need to bear responsibility for this. *The pace of change needs to increase.*

## Medical specialists: flywheel for upscaling

There are plenty of pilot schemes and positive initiatives: from digital consultations between GPs and specialists, telemonitoring of chronically ill patients to the use of robotics and domotica in care of the elderly. Yet upscaling is slow in getting going. Many new initiatives lack sufficient evidence for their effectiveness. They first need to meet the ‘state of science and practice’ before the process of including them in basic insurance policies can be initiated. Telemonitoring and e‑consultations are currently financed in an ad-hoc manner because they do not yet come under the services of the Dutch Healthcare Authority (NZa). Moreover, not all healthcare providers are embracing these innovations, even though they may well be in the best interests of patients. We therefore have a long way to go on all fronts. An important player who can operate as a flywheel here is the medical specialist.

## Where there’s a will there’s a way …

As a health insurer we can also help to improve healthcare, for instance by giving healthcare providers financial capacity to organise their care differently. Or perhaps by conducting independent research so that conclusions can be drawn on the legal criterion of ‘the state of science and practice’. This is how it was possible, in conjunction with the Dutch Cardiology Centres, the Netherlands Society of Cardiology and FocusCura, to include the ‘Hartwacht’ telemonitoring service in health insurance policies.

## The role of e‑Health in chronic heart failure cases

Each year, 20,000 Zilveren Kruis policyholders end up in hospital with chronic heart failure. Total expenses on an annual basis: € 25.5 million. Among this group are 3,800 patients who are actually admitted to hospital; an essential and undesirably intrusive situation for patients. Moreover, clinical admission is expensive; 19% of the patients with chronic heart failure account for 60% of total medical expenses (€ 15.5 million). e‑Health and digitisation of heart care could potentially prevent 30% of these admissions via telemonitoring [2].

## Improved quality, reduced waiting times

As patients send values such as blood pressure and heart rate to a cardiologist via an app, the quality of life is improving considerably for various patient groups (atrial fibrillation, heart failure and hypertension). At the same time, cardiologists obtain detailed insight into the patient’s condition, outliers in the health status of patients are prevented and there are fewer patients visiting A&E.

A number of problems can therefore be tackled at the same time using e‑Health. Waiting times at outpatient departments will be reduced, specialist assistance will be used where really needed and patients will be given a better idea of their own medical conditions. Telemonitoring of chronic heart patients increases productivity and cuts expenses as algorithms take over some of the tasks. The quality of life of patients improves and healthcare is structured more efficiently, while the quality of healthcare improves due to more frequent measurements. This is why it is our ambition to offer this to patients in all regions of the Netherlands.

## Upscaling initiatives, shared responsibility

The policy and parameters have already been developed so nothing now stands in the way of further upscaling. Furthermore, the Dutch Healthcare Authority has relaxed the rules on telemonitoring for next year [3]. The ball is now in the court of the healthcare providers to set to work on this and further integrate e‑Health into the healthcare process. Whichever way you twist or turn it, it will never succeed without the efforts and courage of doctors. They are in a position to offer their patients tomorrow’s care today. I therefore propose that we all work together to ensure we do not keep them waiting on this. Cardiologists of the Netherlands, take on that professional responsibility: who dares to take up the challenge?
